# A Very Compact and Low Profile UWB Planar Antenna with WLAN Band Rejection

**DOI:** 10.1155/2016/3560938

**Published:** 2016-03-21

**Authors:** Avez Syed, Rabah W. Aldhaheri

**Affiliations:** Department of Electrical and Computer Engineering, King Abdulaziz University, Jeddah 21589, Saudi Arabia

## Abstract

A low-cost coplanar waveguide fed compact ultrawideband (UWB) antenna with band rejection characteristics for wireless local area network (WLAN) is proposed. The notch band characteristic is achieved by etching half wavelength C-shaped annular ring slot in the radiating patch. By properly choosing the radius and position of the slot, the notch band can be adjusted and controlled. With an overall size of 18.7 mm × 17.6 mm, the antenna turns out to be one of the smallest UWB antennas with band-notched characteristics. It has a wide fractional bandwidth of 130% (2.9–13.7 GHz) with VSWR < 2 and rejecting IEEE 802.11a and HIPERLAN/2 frequency band of 5.1–5.9 GHz. Stable omnidirectional radiation patterns in the *H* plane with an average gain of 4.4 dBi are obtained. The band-notch mechanism of the proposed antenna is examined by HFSS simulator. A good agreement is found between measured and simulated results indicating that the proposed antenna is well suited for integration into portable devices for UWB applications.

## 1. Introduction

In February 2002, the Federal Communications Commission (FCC) has permitted the use of the frequency band of 3.1 to 10.6 GHz for unlicensed wireless communication [1]. Hence, the ultrawideband (UWB) technology is used in modern radio communication systems for transmitting data over transmission channels. The antennas are the key and challenging element of UWB systems which use 3.1 to 10.6 GHz frequency band. Therefore, more attention and research effort are made in designing UWB antenna as it provides higher and better data rate over a large bandwidth. However, in the designated bandwidth of UWB system, there are some other existing narrow band systems that occupy the UWB spectrum, such as WLAN IEEE 802.11a and HIPERLAN/2 operating in the 5.15–5.825 GHz band. The interference from such narrow bands hinders the performance of UWB systems. Hence, several UWB antennas with band notch function are developed by using conventional techniques to avoid interference in UWB communication systems [2–9]. One of the methods of achieving the band stop characteristics is by inserting slots of various shapes and sizes into the radiating patch or the feed line or the ground plane [2–5]. A mushroom-type electromagnetic band gap (EBG) structures are used in [6] to prevent interference from unwanted narrow bands. In [7], the band notch characteristics are obtained by using a slot type split ring resonator. The antenna reported in [8] consists of a square slot in the radiating patch. The WLAN notch operation is obtained by vertically placing the rectangular coupling strip in the centre of the slot patch. In [9], the band rejection is achieved by attaching two parasitic patches to the bottom layer of the antenna. However, most of these designs have a large size [4–9] and complex geometry [2, 6, 9]. The antennas reported in [5, 7] fail to suppress the interference from the entire WLAN band (5.15–5.825 GHz). The proposed UWB antenna is smaller in size than the antennas reported in [3–9] with very simple geometry.

In this paper, a low-cost, very simple, and compact CPW fed UWB antenna with band notch for WLAN is proposed. The proposed antenna operates from 2.9 to 13.7 GHz with a rejected frequency band of 5.1–5.9 GHz. The notch band function is obtained by etching a C-shaped annular ring slot in the radiating patch. The antenna parameters are optimized using the Ansoft HFSS electromagnetic simulator. The paper is organized as follows: Section 2 gives the basic design of the proposed UWB antenna with band rejection. In Section 3, the parametric analysis is presented.  Section 4 presents the simulated and measured results and comparison of the proposed antenna with other antennas in literature and finally Section 5 concludes the presented work.

## 2. Antenna Structure and Design

**Figure 1:**
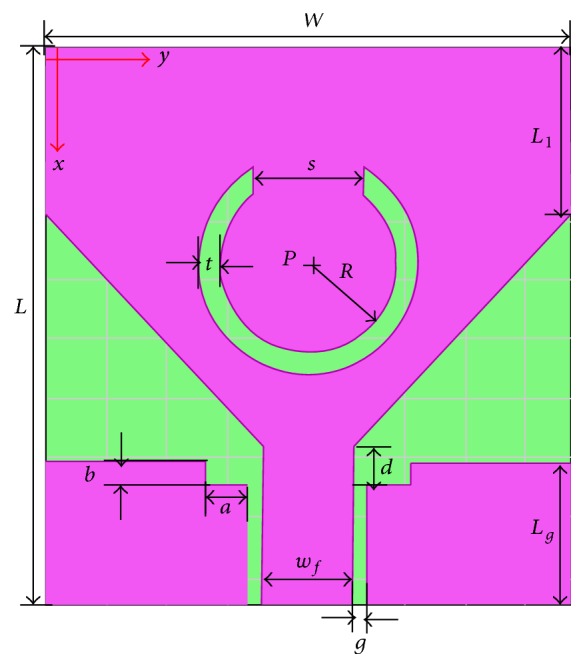
Geometry of the proposed antenna.

**Figure 2:**
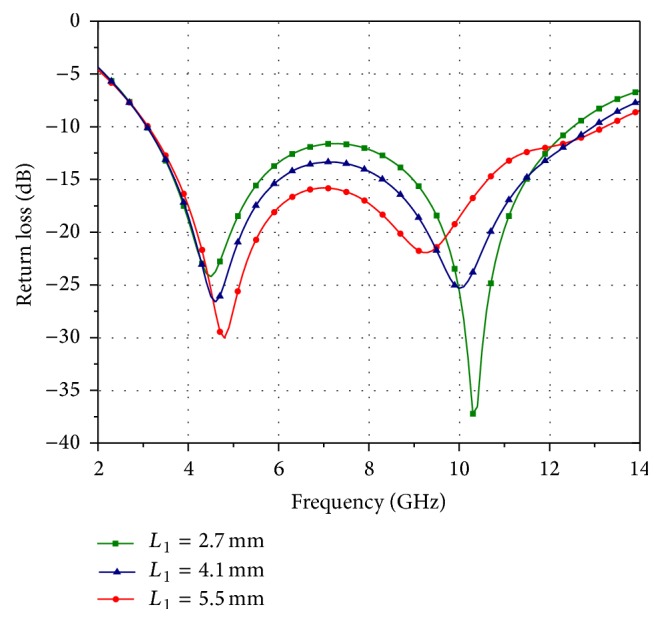
Return loss of the UWB antenna (without slot) for different values of *L*_1_.

The geometry of the proposed WLAN band-notched UWB antenna is shown in Figure 1. The proposed antenna is printed on an inexpensive FR4 substrate with a dielectric permittivity of 4.4. The thickness of the substrate is 1.5 mm and the overall dimension of the designed antenna is 18.7 mm × 17.6 mm which is one of the smallest UWB antennas with band-notched characteristics. The compact small size with a metallic layer on one side of the substrate makes the antenna easily integrate with system circuits. The antenna consists of a bevel radiating patch and a modified ground plane, which are responsible for a large impedance bandwidth. The angle of the bevel is controlled by the dimension *L*_1_. Hence, the entire band can be enhanced by adjusting the length *L*_1_.  Figure 2 shows the return loss of the UWB antenna (without slot) for different values of *L*_1_. As illustrated in Figure 2, by varying the length *L*_1_, the impedance matching and the impedance bandwidth are affected for the entire frequency range. The length *L*_1_ is optimized for 5.5 mm using HFSS and a change in this value leads to poor impedance matching. The stopband function is achieved by etching a C-shaped annular ring slot in the radiating patch. The annular slot has strong coupling with the radiating patch which helps to reject the WLAN band without any change in ultrawide bandwidth.

**Figure 3:**
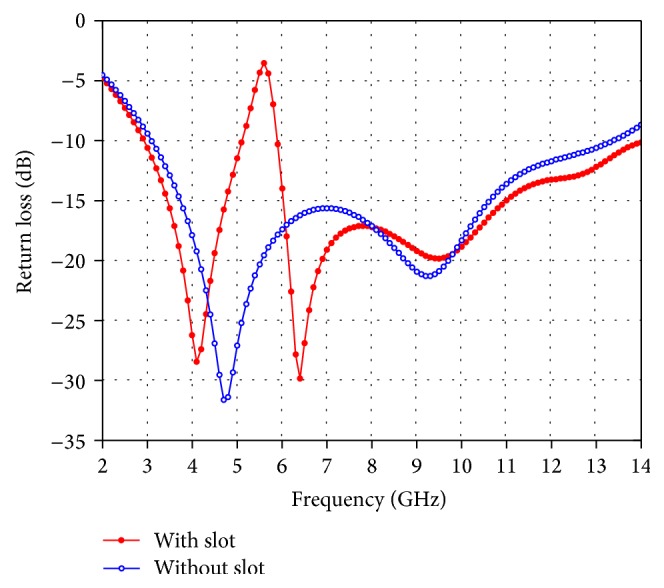
Return loss of the proposed antenna with and without slot.

The stopband frequency generated by the C-shaped slot can be formulated as follows [13]:

(1) $$\begin{array}{c} f_{stop}=\frac{C}{2L_{s}\sqrt{\varepsilon_{eff}}}, \end{array}$$

where *L*_*s*_ is the total length of the C-shaped slot, *C* is the speed of light, and *ε*_eff_ is the effective dielectric constant which is given by (*ε*_*r*_ + 1)/2. Here *ε*_*r*_ is the dielectric permittivity of the substrate. The total length of the slot is given by *L*_*s*_ = 2*πR* + 2*t* − *s*. The centre rejecting frequency, *f*_stop_ = 5.5 GHz, is obtained when the total slot length is set to *L*_*s*_ which is practically equal to 0.5*λ*_gs_. Here *λ*_gs_ is guided wavelength and is equal to $\lambda_{0}/\sqrt{\varepsilon_{eff}}$, where *λ*_0_ = *C*/*f*_stop_. A 50 Ω coplanar waveguide (CPW) feed line is used to feed the radiating element. The Ansoft HFSS electromagnetic simulator has been used to simulate and optimize the proposed antenna structure. The optimized parameters are as follows: *L* = 18.7 mm, *W* = 17.6 mm, *w*_*f*_ = 3 mm, *L*_*g*_ = 4.85 mm, *L*_1_ = 5.5 mm, *g* = 0.5 mm, *d* = 1.3 mm, *a* = 1.4 mm, *b* = 0.75 mm, *t* = 0.75 mm, *s* = 3.6 mm, and *R* = 2.95 mm.  Figure 3 shows the return loss of the proposed antenna with and without a slot. It is seen that, without insertion of the slot, the proposed antenna covers entire UWB, while the inclusion of slot introduces a notched band within the pass band. The impedance characteristics of the proposed antenna with slot are similar to those of the antenna without a slot.

## 3. Parametric Analysis

**Figure 4:**
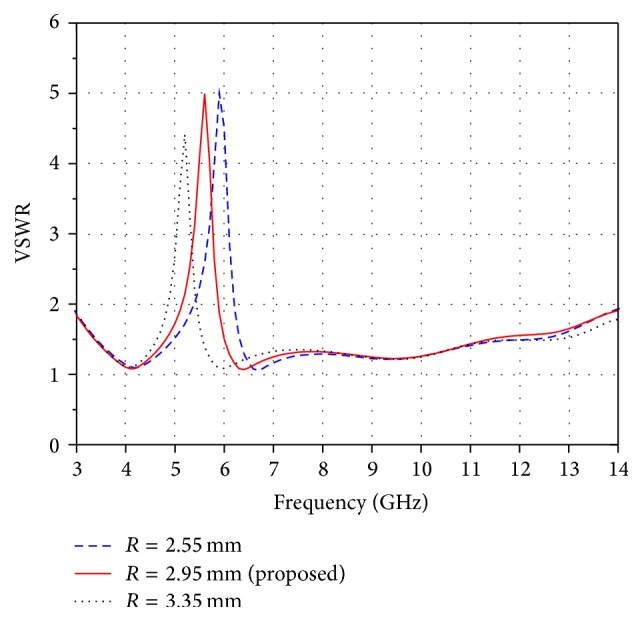
Effect of parameter *R* on band notch function.

Results shown in Figure 4 indicate that the notch band characteristic is controllable by varying the radius *R*. It is observed that the centre frequency of the notch band moves to the lower frequency region when the radius *R* is varied from 2.55 mm to 3.35 mm. In this design, a better band stop feature is obtained at an optimized value of *R* = 2.95 mm which rejects the whole WLAN band. It is seen that the bandwidth of notch band is decreased when *R* is other than 2.95 mm.

**Figure 5:**
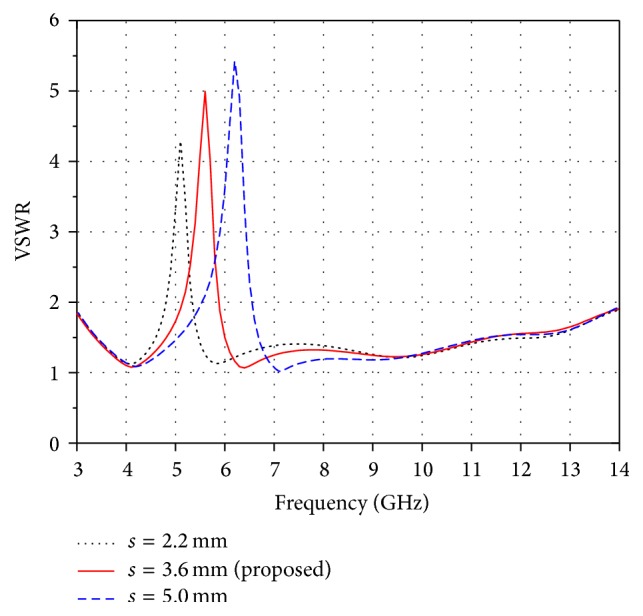
Effect of parameter *s* on band notch function.

Figure 5 shows the effect of parameter, *s*, on band notch function. It is seen that the centre frequency of notch band is shifted towards higher frequency band as the value of *s* is increased from 2.2 mm to 5.0 mm. Hence, the parameter *s* is optimized to obtain better notch results. The optimized values of *s* and *R* at which the proposed antenna rejects the whole WLAN band with excellent notch features are 3.6 mm and 2.95 mm, respectively. The inclusion of slot just creates a notched band feature without affecting the impedance characteristics of the proposed antenna.

**Figure 6:**
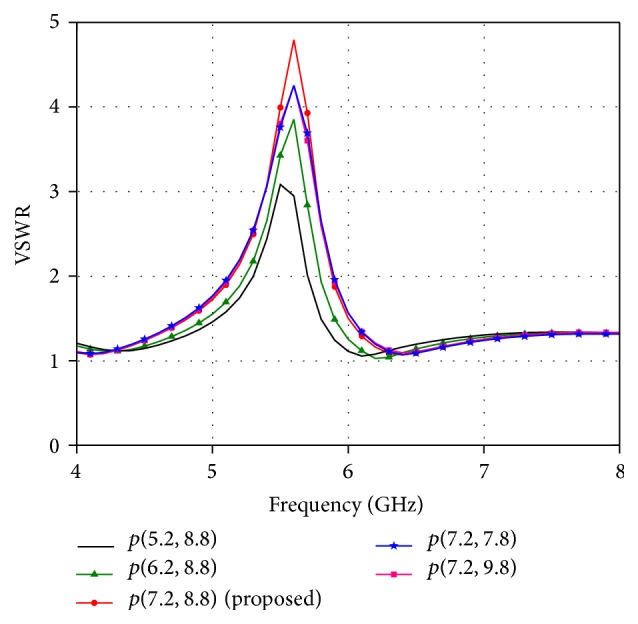
Effect of location of slot on band notch function.

The location of the C-shaped slot is controlled by the parameter *p* which is the centre of ring slot. The effect of varying the slot position (*p*) while keeping other parameters constant is presented in Figure 6. The proposed antenna covers the entire UWB bandwidth irrespective of slot position *p*. The parameter *p* affects only the notched band and hence for simplicity and better visuality only the variation in the notched band is shown in Figure 6. The better notch band characteristics are obtained when the slot is located at *p*(7.2,8.8). It is observed that the notch bandwidth decreases with the change in the slot position from (7.2,8.8) which in turn fails to reject the whole WLAN band (5.15–5.825 GHz).

## 4. Results and Discussions

**Figure 7:**
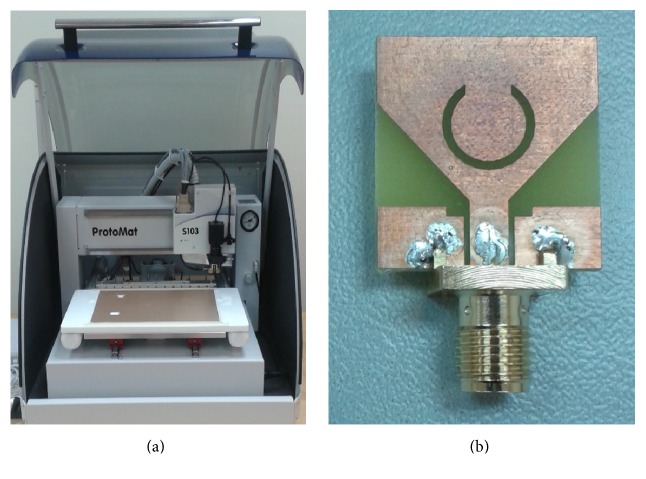
(a) LPKF machine (S103) and (b) proposed antenna prototype.

**Figure 8:**
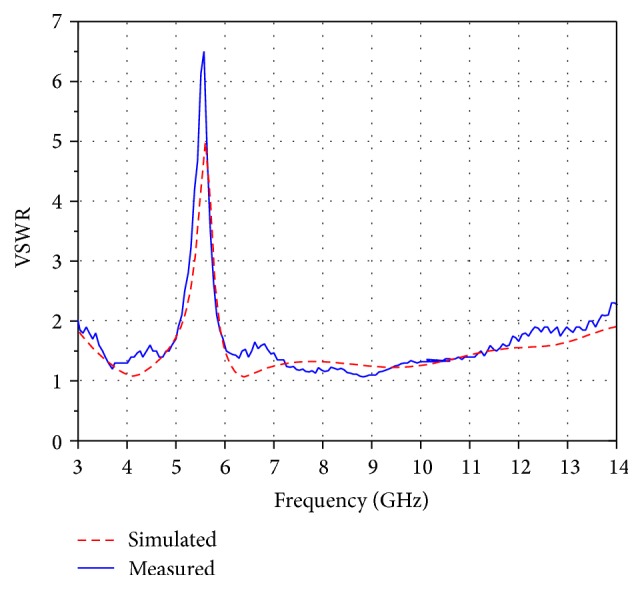
Simulated and measured VSWR curves.

**Figure 9:**
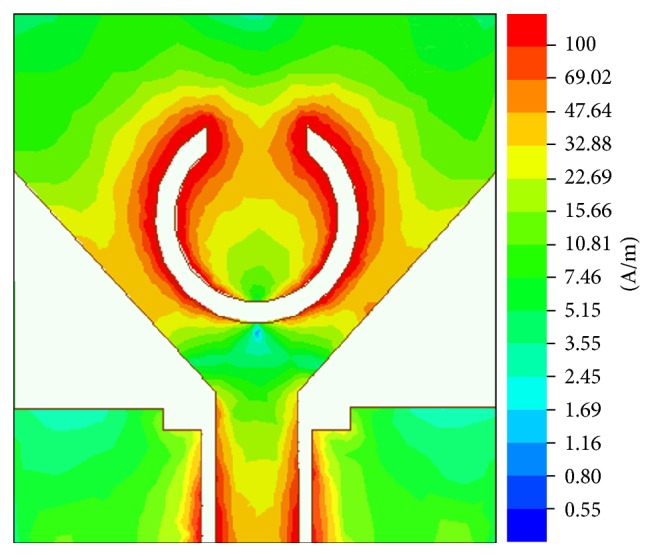
Surface current distribution on the antenna at 5.5 GHz.

The Ansoft HFSS version 15 simulator is employed to simulate the proposed antenna. The antenna with the optimized parameters was fabricated using PCB LPKF (S103) prototyping machine, which is shown in Figure 7(a), and the performance was measured using Agilent N5225A PNA network analyzer. The fabricated antenna prototype for experimental measurements is as shown in Figure 7(b). The simulated and measured results of the VSWR for the proposed design are shown in Figure 8. Good agreement has been observed between the simulated and measured results. The measured result shows that the operating frequency band of the proposed antenna ranges from 2.9 GHz to 13.7 GHz (VSWR < 2) which covers the entire UWB while rejecting the 5.1–5.9 GHz band. A slight deviation in results is mainly due to fabrication tolerance and the effect of SMA connector. To further investigate the band-notched function, the surface current distribution of the designed antenna at the centre rejected frequency has been simulated and shown in Figure 9. It can be seen that the current concentrated on the inner and outer edges of C slot at 5.5 GHz. Hence, attenuation is produced at the notch frequency which successfully rejects 5.1–5.9 GHz frequency band which in turn prevents the potential interference from WLAN band.

The proposed UWB antenna with notch characteristics has very simple, compact size and is easy to fabricate on a low-cost substrate. The performance of proposed work in terms of the size of the antenna, impedance bandwidth, and notch characteristics is compared with other existing antennas in the literature [5, 6, 8, 10–12] as mentioned in Table 1. From the comparison, it can be concluded that the proposed antenna structure gives better impedance bandwidth and excellent notch characteristics with relatively very small and simple structure.

**Figure 10:**
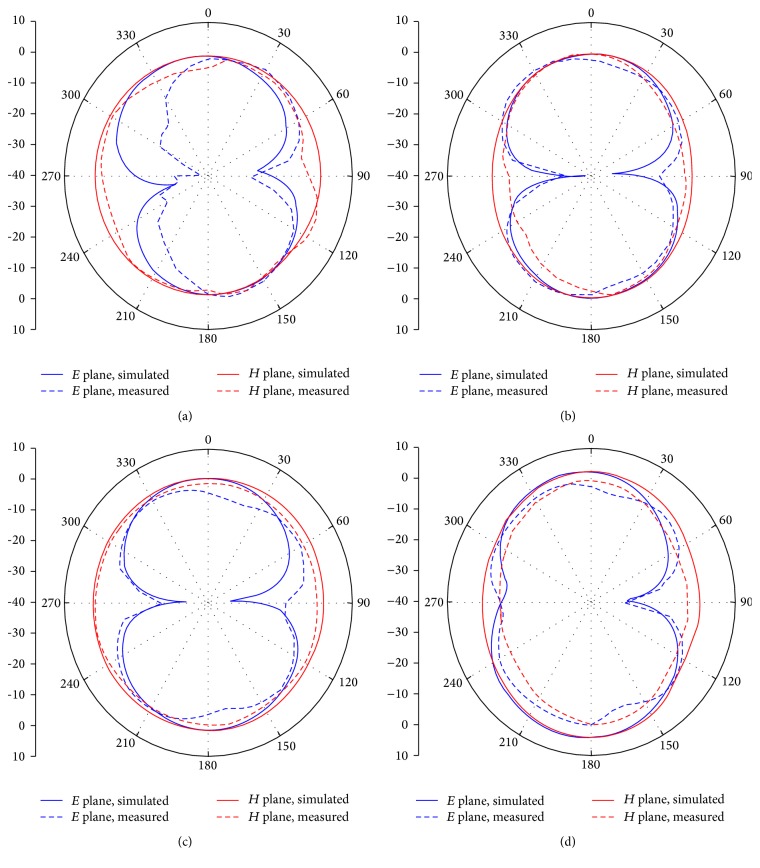
Measured (dash line) and simulated (solid line) radiation patterns of the proposed antenna at (a) 4 GHz, (b) 5.5 GHz, (c) 6.4 GHz, and (d) 10 GHz.

**Figure 11:**
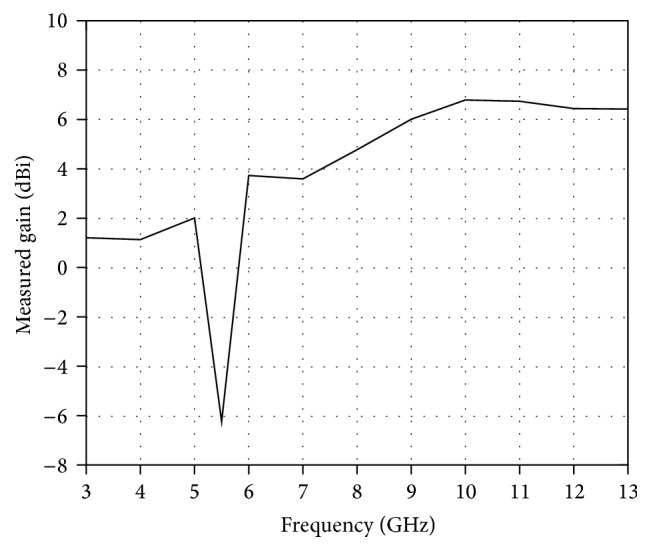
Measured gain of the proposed antenna.

The measured and simulated *E* plane and *H* plane radiation patterns of the proposed antenna at 4.0 GHz, 5.5 GHz, 6.4 GHz, and 10 GHz are presented in Figure 10. It is seen from the results that the antenna exhibits an omnidirectional and a stable radiation pattern in *H* plane. In *E* plane, the radiation patterns are slightly bidirectional with two nulls and the patterns are similar to that of the wideband monopole antenna. Overall the patterns are stable in the entire operation band making the antenna a strong candidate for UWB applications.  Figure 11 presents the measured gain of the proposed antenna. The average antenna gain is about 4.4 dBi over most of the operating band. However, the antenna shows a sharp gain decrease in 5.1–5.9 GHz band. This result shows that the antenna is successfully performed with the rejection in WLAN band.

## 5. Conclusions

In this paper, a very simple, low-cost, and compact UWB antenna with band stop characteristics has been proposed and implemented. The fabricated prototype has the frequency band of 2.9 GHz to 13.7 GHz with a rejection band around 5.1–5.9 GHz, which is due to the cutting of a C-shaped slot in the radiating patch. The notch band can be tuned and adjusted by properly choosing the radius and the location of the slot. With a compact size of 18.7 mm × 17.6 mm, it turns out to be one of the smallest UWB antennas which can be easily integrated into system circuits. The low profile and stable radiation characteristics of the proposed antenna make it suitable for being used in UWB applications.

## Figures and Tables

**Table 1:** Comparison of the proposed antenna with the existing antennas in literature. Here *λ*_*g*_ is the guided wavelength at the first resonance.

Reference number	Dimensions	Impedance bandwidth (−10 dB criteria)	Notched band (GHz)
[5]	35 mm × 35 mm 0.73*λ*_*g*_ × 0.73*λ*_*g*_	117%	4.80–5.70

[6]	39 mm × 35 mm 0.94*λ*_*g*_ × 0.84*λ*_*g*_	109%	5.15–5.85

[8]	35 mm × 30 mm 0.75*λ*_*g*_ × 0.64*λ*_*g*_	114%	5.12–6.08

[10]	30 mm × 39.3 mm 0.49*λ*_*g*_ × 0.64*λ*_*g*_	129%	5.15–5.825

[11]	36 mm × 30 mm 0.66*λ*_*g*_ × 0.55*λ*_*g*_	132%	4.85–6.04

[12]	35 mm × 35 mm 0.83*λ*_*g*_ × 0.83*λ*_*g*_	109%	5.00–6.00

**Proposed antenna**	**18.7 mm × 17.6 mm** **0.45** ***λ***_**g**_ ** × 0.42** ***λ***_**g**_	**130%**	**5.10–5.90**
